# Repression of transcription factor AP-2 alpha by PPARγ reveals a novel transcriptional circuit in basal-squamous bladder cancer

**DOI:** 10.1038/s41389-019-0178-3

**Published:** 2019-11-26

**Authors:** Hironobu Yamashita, Yuka I. Kawasawa, Lauren Shuman, Zongyu Zheng, Truc Tran, Vonn Walter, Joshua I. Warrick, Guoli Chen, Hikmat Al-Ahmadie, Matthew Kaag, Pak Kin Wong, Jay D. Raman, David J. DeGraff

**Affiliations:** 1 Department of Pathology and Laboratory Medicine, Pennsylvania State University College of Medicine, Hershey, PA USA; 2 Institute for Personalized Medicine, Pennsylvania State University, Hershey, PA USA; 3 Department of Pharmacology, Pennsylvania State University, Hershey, PA USA; 4 Department of Biochemistry and Molecular Biology, Pennsylvania State University College of Medicine, Hershey, PA USA; 5 Department of Surgery, Division of Urology, Pennsylvania State University College of Medicine, Hershey, PA USA; 6 Department of Public Health Sciences, Pennsylvania State University College of Medicine, Hershey, PA USA; 7 Department of Pathology, Memorial Sloan Kettering Cancer Center, New York, NY USA; 8 Department of Biomedical Engineering, Pennsylvania State University, University Park, PA USA

**Keywords:** Bladder cancer, Nuclear receptors

## Abstract

The discovery of bladder cancer transcriptional subtypes provides an opportunity to identify high risk patients, and tailor disease management. Recent studies suggest tumor heterogeneity contributes to regional differences in molecular subtype within the tumor, as well as during progression and following treatment. Nonetheless, the transcriptional drivers of the aggressive basal-squamous subtype remain unidentified. As PPARɣ has been repeatedly implicated in the luminal subtype of bladder cancer, we hypothesized inactivation of this transcriptional master regulator during progression results in increased expression of basal-squamous specific transcription factors (TFs) which act to drive aggressive behavior. We initiated a pharmacologic and RNA-seq-based screen to identify PPARɣ-repressed, basal-squamous specific TFs. Hierarchical clustering of RNA-seq data following treatment of three human bladder cancer cells with a PPARɣ agonist identified a number of TFs regulated by PPARɣ activation, several of which are implicated in urothelial and squamous differentiation. One PPARɣ-repressed TF implicated in squamous differentiation identified is Transcription Factor Activating Protein 2 alpha (TFAP2A). We show TFAP2A and its paralog TFAP2C are overexpressed in basal-squamous bladder cancer and in squamous areas of cystectomy samples, and that overexpression is associated with increased lymph node metastasis and distant recurrence, respectively. Biochemical analysis confirmed the ability of PPARɣ activation to repress TFAP2A, while PPARɣ antagonist and PPARɣ siRNA knockdown studies indicate the requirement of a functional receptor. In vivo tissue recombination studies show TFAP2A and TFAP2C promote tumor growth in line with the aggressive nature of basal-squamous bladder cancer. Our findings suggest PPARɣ inactivation, as well as TFAP2A and TFAP2C overexpression cooperate with other TFs to promote the basal-squamous transition during tumor progression.

## Introduction

While the most commonly diagnosed type of bladder cancer (BC) is morphologically defined as urothelial carcinoma, the existence of morphologic variants of BC and their association with clinical outcomes has been recognized for decades. Molecular studies show that variant morphologies in BC exhibit unique gene expression patterns, which may contribute to differing oncologic outcomes in these patients^1,2^. Moreover, recent studies have identified a striking degree of intratumoral, morphologic and molecular heterogeneity in advanced BC^3^. If advanced BC with intratumoral heterogeneity is largely clonal in nature, the fact that the vast majority of carcinoma in situ (considered the precursor for the majority of advanced BC) lesions are luminal^4^ strongly suggests molecular subtype is “plastic” and can evolve over time. This perspective is substantiated by the fact that areas of variant morphology exhibit significant differences in gene expression subtype within a single tumor, yet harbor a large number of identical genetic alterations^5^.

While the exact temporal sequence of genetic alterations in BC and how these alterations directly contribute to tumor heterogeneity is unknown, several lines of evidence implicate the steroid hormone receptor peroxisome proliferator active receptor gamma (PPARɣ) in this process. For example, activation of this nuclear hormone receptor has been shown to oppose squamous differentiation (SqD) in vitro^6^, while inactivation of both PPARɣ and PTEN expression drive squamous changes in vivo^7^. Moreover, PPARɣ is amplified at the genomic level in the luminal BC subtype where it is consistently overexpressed^8–10^, and activation of PPARɣ cooperates with overexpression of FOXA1 and GATA3 to “reprogram” the basal-squamous cell line 5637 to exhibit a luminal expression pattern^11^. While these observations suggest PPARɣ is a master regulator of luminal BC cell fate, as well as a potential therapeutic target^8,12–14^, the transcriptional mediators of basal-squamous BC remain unidentified.

Accordingly, we hypothesized PPARɣ actively represses transcription factors (TFs) that drive basal-squamous gene expression in human BC, and by extension, inactivation of PPARɣ drives expansion of basal-squamous clones by upregulating these TFs. We tested the initial component of this hypothesis in the current study by performing a pharmacologic and RNA-seq based screen to identify PPARɣ-repressed TFs operative in driving the basal-squamous subtype. In doing so, we provide the first evidence identifying members of the Transcription Factor Activator Protein 2 (TFAP2) family as markers of basal-squamous BC that play a direct role in mediating the phenotype of this aggressive subtype of disease.

## Results

### PPARɣ is a master regulator of luminal gene expression in bladder cancer cells

**Fig. 1:**
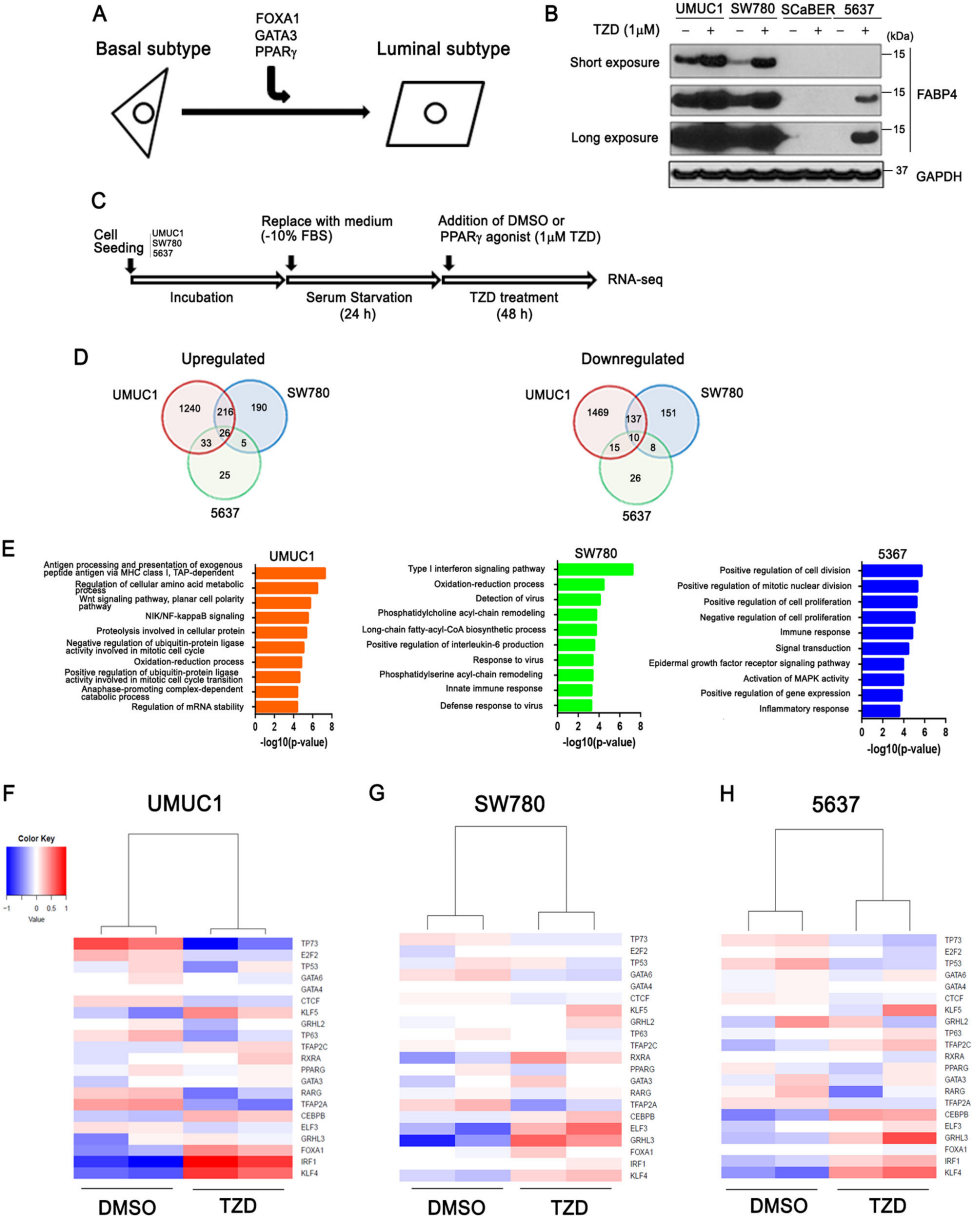
Identification of PPARɣ-repressed transcription factors. **a** Our previous study^11^ shows FOXA1, GATA3, and PPARɣ activation cooperate to “reprogram” a human basal BC cell line (5637) to exhibit a luminal gene expression subtype, thus providing a platform for the identification of PPARɣ-regulated genes. **b** Western blot analysis of FABP4 in UMUC1, SW780, SCaBER, 5637 cell after 48 h PPARɣ agonist (Rosiglitazone (TZD)) treatment. Short and long exposure of FABP4 were also shown. **c** Experimental design for PPARɣ activation via TZD treatment in BC cells (see materials and methods). **d** Venn diagram showing shared and unique sets of significantly upregulated (left) or downregulated (right) genes following 48 h TZD treatment of UMUC1, SW780 and 5637 cells. **e** GO analysis based on significantly upregulated genes following PPARɣ activation. Biological process by GO analysis based on significantly upregulated genes following PPARɣ activation in UMUC1, SW780, and 5637 cells. Top 10 biological process cetegories of significantly upregulated by PPARɣ activation are shown. The vertical axis shows biological process cetegories, and horizontal axis shows the –log10(*P*-value). **f**–**h** Heatmap shows hierarchical clustering of RNA-seq data following treatment of UMUC1 (**f**), SW780 (**g**), 5637 (**h**) with vehicle control (DMSO) or TZD for 48 h. Genes for clustering analysis based on previous studies suggesting a role for urothelial differentiation and/or differential expression in BC molecular subtypes are shown. Expression values are median centered by gene in all heatmap displays.

In a previous study^11^, we reported that overexpression of FOXA1 and GATA3 cooperated with PPARɣ activation to “reprogram” a basal-squamous BC cell line (5637), resulting in the activation of a luminal molecular signature (Fig. 1a). Although no single factor in these experiments was able to reprogram 5637, the largest shift in gene expression during these experiments followed PPARɣ activation with the agonist rosiglitazone (TZD). Based on this observation, we hypothesized the existence of a set of basal-squamous-specific TFs that are repressed in the presence of active PPARɣ signaling. In an effort to identify PPARɣ-regulated TFs, we first screened six common, PPARɣ positive^11^ BC cell lines (UMUC1, SW780, SCaBER, 5637, HT1376, and HT1197) for responsiveness to the PPARɣ agonist TZD. Western blotting analysis for the putative PPARɣ response gene fatty acid binding protein 4 (FABP4) following 48 h of TZD treatment identified UMUC1, SW780, and 5637 cell as PPARɣ-responsive (Fig. 1b), while HT1376, HT1197 did not show FABP4 expression (Supplementary Fig. S1). UMUC1, SW780, and 5637 cells were subsequently used for RNA-Seq studies to identify PPARɣ-regulated TFs (Fig. 1c). While this approach identified a number of cell line-specific TZD-regulated genes following treatment, we additionally identified a total of 26 and 10 genes coordinately upregulated and downregulated amongst 5637, UMUC1, and SW780 following PPARɣ activation, respectively (Fig. 1d, Supplementary Table S1). Consistent with previous reports indicating a central role for PPARɣ in the BC cell autonomous regulation of cytokine production^8^, Gene Ontology (GO) enrichment analysis (Fig. 1e) identified some immune related pathways as being associated with genes upregulated following TZD treatment. While GO enrhichment analysis of genes which decreased following TZD treatment identified alterations in some immune pathways in SW780, pathways associated with TZD treatment of UMUC1 and 5637 were associated largely with cell growth and gene expression (Supplementary Fig. S2). As maintenance of a given molecular subtype in BC most likely results from the activity of a small subset of transcriptional master regulators^15^, we further examined our RNA-seq results in an effort to identify altered expression of key TFs implicated in urothelial differentiation following PPARɣ activation. Individual hierarchical clustering of RNA-seq data from UMUC1 (Fig. 1f), SW780 (Fig. 1g), and 5637 (Fig. 1h) identified transcription factor AP-2 alpha (TFAP2A) and CCCTC binding factor (CTCF) as TZD-repressed transcription factors in all three cell lines, while expression of the pluripotency factor Kruppel-like factor 4 (KLF4) was increased by TZD treatment in all three cell lines (Fig. 1f–h and Supplementary Fig. S3). Our findings are in agreement with previous reports indicating a role for CTCF^16^ and KLF4^17^ in urothelial differentiation and BC, respectively, and additionally suggest a role for TFAP2A in the emergence of a basal-squamous phenotype following PPARɣ inactivation in BC.

### PPARɣ signaling represses TFAP2A expression in bladder cancer cells

**Fig. 2:**
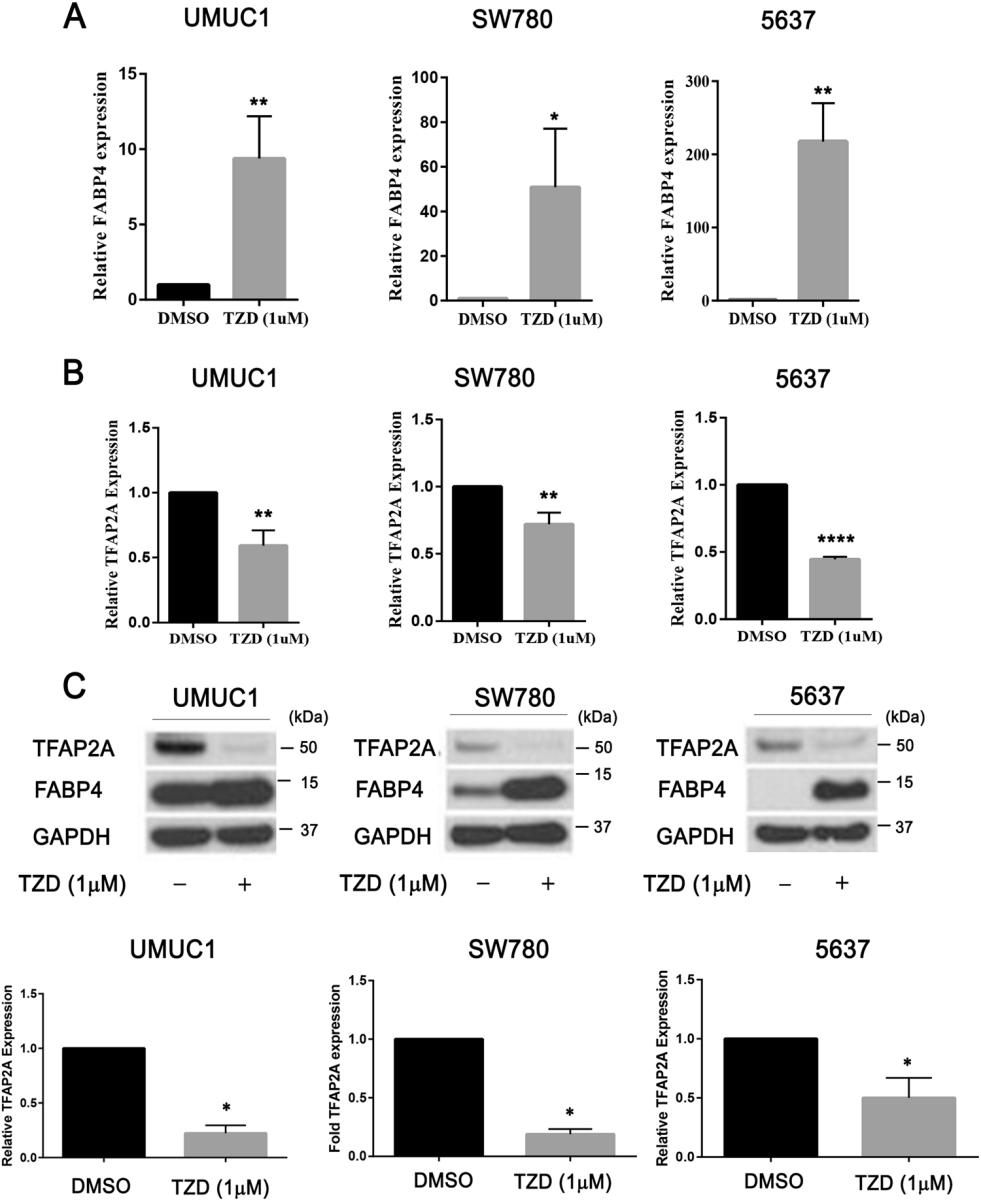
PPARɣ activation represses TFAP2A expression in human bladder cancer cells. **a** q-RT-PCR analysis of FABP4 expression levels in UMUC1, SW780, and 5637 cells after 48 h PPARɣ agonist treatment. FABP4 expression was normalized by 18S ribosomal RNA, internal control. Data are expressed as the mean ± S.D. from three independent experiments. *p < 0.05, **p < 0.01 (Student’s *t* test). **b** q-RT-PCR analysis of TFAP2A expression levels in UMUC1, SW780 and 5637 cells after 48 h PPARɣ agonist treatment. TFAP2A expression was normalized by 18S ribosomal RNA, internal control. Data are expressed as the mean ± S.D. from three independent experiments. **p < 0.01, ****p < 0.0001 (Student’s *t* test). **c** Western blot analysis of TFAP2A protein expression level in UMUC1, SW780, 5637 cells after 48 h PPARɣ agonist treatment. Densitometric analysis of western blot of TFAP2A expression (below). In **a**–**c**, relative expression levels of FABP4 and TFAP2A mRNA and or/protein after PPARɣ agonist treatment are represented compared with that of DMSO treatment. Data are expressed as the mean ± S.D. from three independent experiments. *p < 0.05 (Student’s *t* test).

**Fig. 3:**
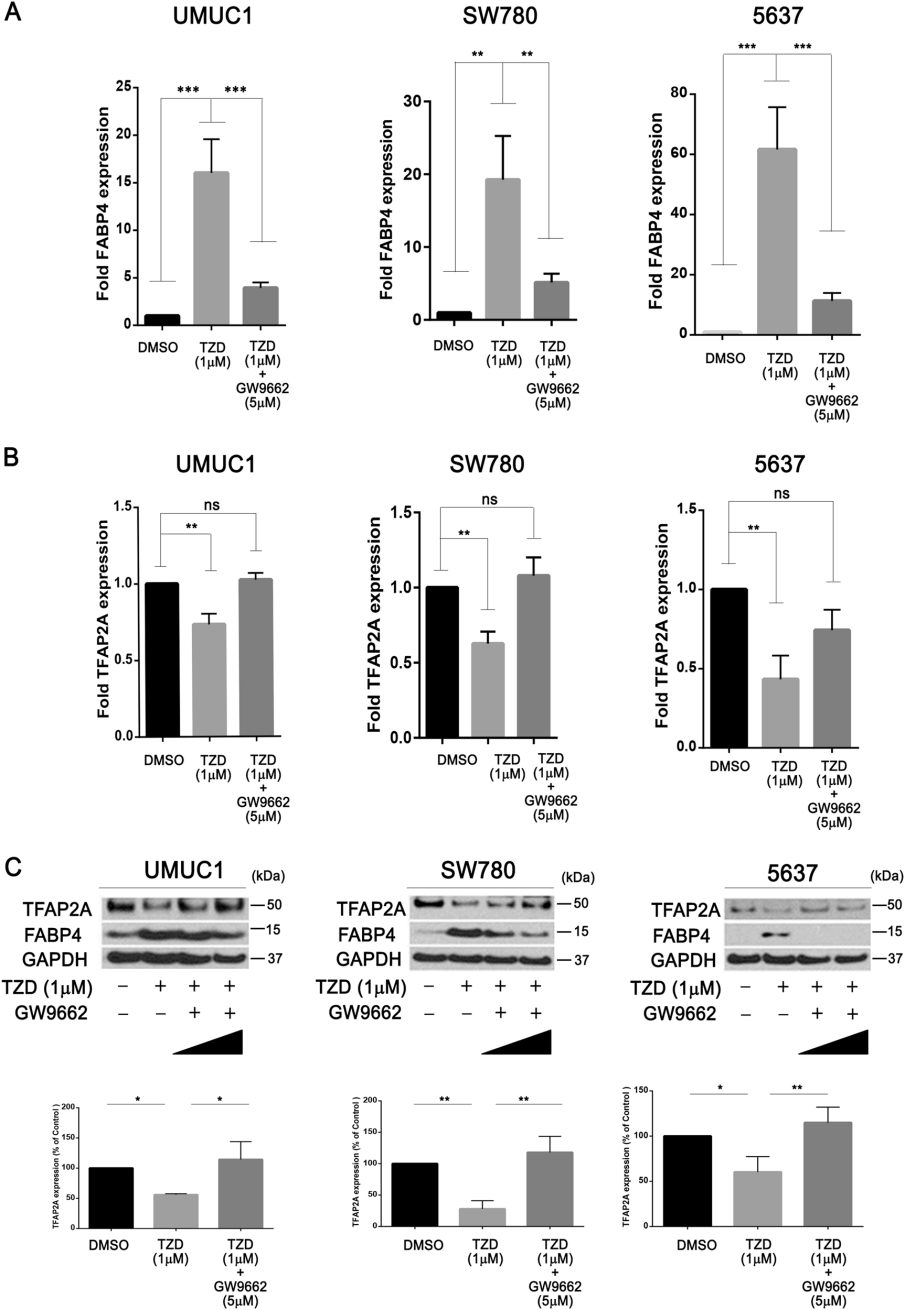
Repression of TFAP2A expression via PPARɣ is dependent on a functional receptor. **a**, **b** q-RT-PCR analysis of FABP4 (**a**) and TFAP2A (**b**) expression levels in UMUC1, SW780, and 5637 cells after PPARɣ agonist (1 μM) treatment alone or in the presence of the PPARɣ antagonist (5 μM), GW9662. The putative PPARɣ-regulated gene, FABP4 was used as positive control for drug treatments. Relative expression levels of TFAP2A and FABP4 after PPARɣ treatment is represented relative to that of DMSO treatment. Data are expressed as the mean ± S.D. from three independent experiments. **p < 0.01, ***p < 0.001, ns: not significant, one-way ANOVA with post hoc multiple comparison (Tukey). **c** Western blotting analysis of TFAP2A protein expression levels in UMUC1, SW780, and 5637 cells after PPARɣ agonist (1 μM) treatment alone and in the presence (1 μM and 5 μM) of the PPARɣ antagonist, GW9662. Densitometric analysis of western blotting of TFAP2A expression (below). TFAP2A expression was normalized by GAPDH, internal control. Data are expressed as the mean ± S.D. from three independent experiments. *p < 0.05, **p < 0.01, one-way ANOVA with post hoc multiple comparison (Tukey).

We next investigated the regulatory relationship between PPARɣ and TFAP2A. Treatment of 5637, UMUC1 and SW780 with 1 µM TZD for 48 h followed by Q-RT-PCR for FABP4 confirmed responsiveness of these lines to PPARɣ activation (Fig. 2a). Q-RT-PCR and western blotting confirmed the ability of PPARɣ activation to repress TFAP2A expression at the mRNA (Fig. 2b) and protein (Fig. 2c) levels, respectively. To confirm a role for a functional PPARɣ receptor, we performed identical TZD treatments of 5637, UMUC1 and SW780 alone and in conjunction with the PPARɣ antagonist GW9662. Q-RT-PCR results show that while TZD treatment significantly increased FABP4 mRNA expression and decreased TFAP2A mRNA expression, these significant changes were abolished in the presence of GW9662 co-treatment (Fig. 3a, b). In addition, while TZD treatment significantly reduced TFAP2A protein levels, this was prevented following co-treatment with GW9662 treatment (Fig. 3c). Furthermore, PPARɣ siRNA-mediated knockdown in UMUC1 prevented rosiglitazone-mediated reductions in TFAP2A mRNA and protein expression, as well as increased FABP4 mRNA and protein (Supplementary Fig. S4) These results suggests that TZD-induced repression of TFAP2A mRNA and protein requires a functional PPARɣ receptor in BC cells.

### TFAP2A and TFAP2C expression are markers of basal-squamous bladder cancer

**Fig. 4:**
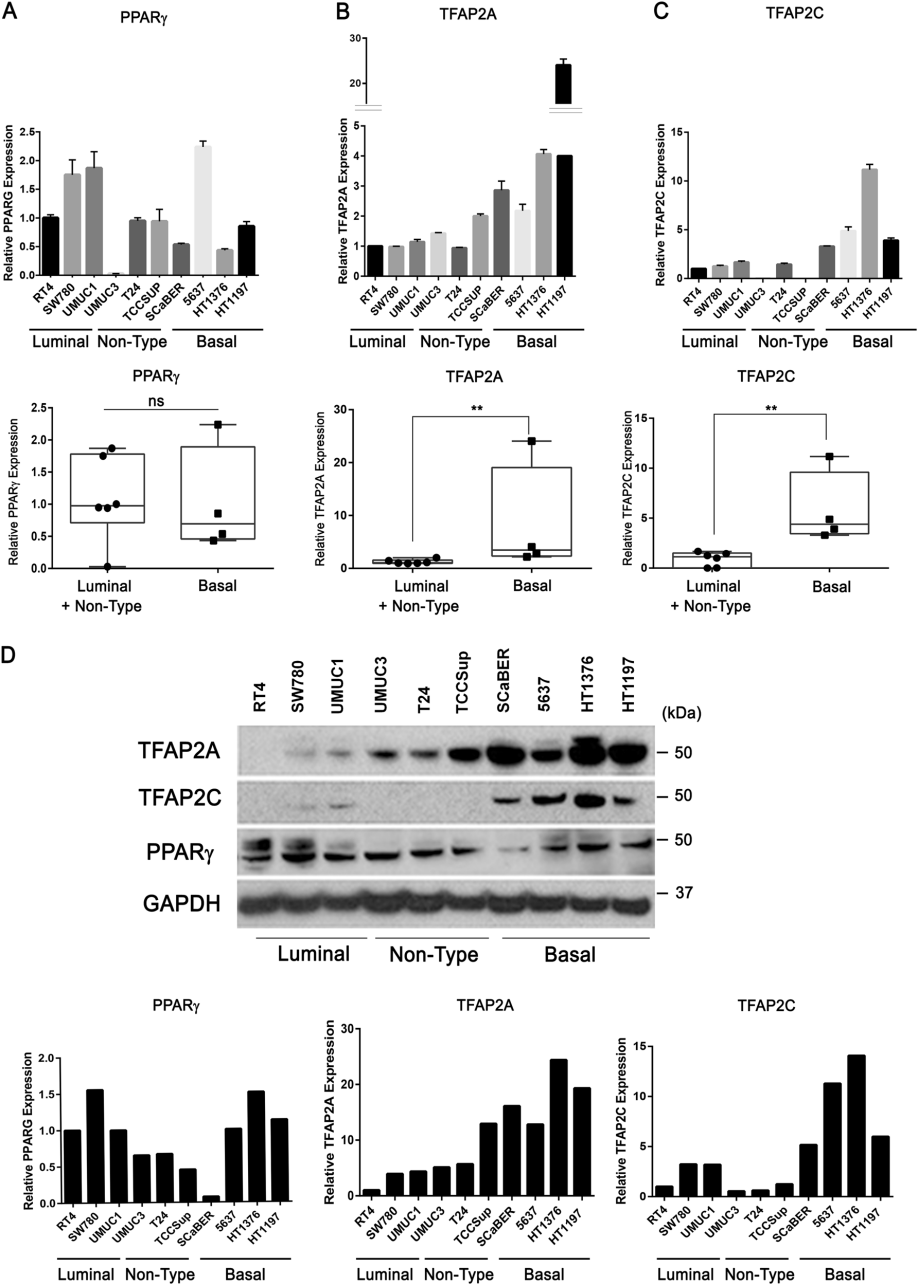
TFAP2A and TFAP2C are highly expressed in basal-squamous bladder cancer cell lines. **a–c** q-RT-PCR analysis of mRNA expression of PPARɣ (**a**), TFAP2A (**b**), and TFAP2C (**c**) in 10 human BC cell lines. Data are expressed as the mean ± S.D. from three independent experiments. Data of Luminal/Non-Type vs Basal are expressed as the medians ± S.D. **p < 0.01, ns: not significant, Mann–Whitney U test. **d** Western blot analysis of TFAP2A, TFAP2C, and PPARɣ protein expression in 10 human BC cell lines (Luminal: RT4, SW780, UMUC1/ Non-type: UMUC3, T24, TCCSup/Basal: SCaBER, 5637, HT1376, HT1197). Densitometric analysis of western blotting data is below. TFAP2A, TFAP2C, and PPARɣ expression was normalized by GAPDH, internal control.

Basal-squamous BC is significantly enriched for SqD, and several studies have implicated TFAP2A and its paralog TFAP2C in development and differentiation of normal squamous epithelium^18–21^. Therefore, our identification of TFAP2A as a PPARɣ-repressed transcriptional regulator suggested a role for members of the TFAP2 family in basal-squamous BC. First, we utilized q-RT-PCR to examine the expression of TFAP2A, TFAP2C and PPARɣ in a panel of BC cell lines representative of luminal and basal-squamous BC, as well as a group of cell lines which does not fit under either gene expression subtype (“non-type”). PPARɣ expression was not correlated with cell line gene expression subtype (Fig. 4a), nor significantly associated with the expression of TFAP2A and TFAP2C (data not shown). However, PPARG expression significantly exhibited negative correlation with TFAP2A, not TFAP2C (Supplementary Fig. S5). On the other hand, TFAP2A (Fig. 4b) and TFAP2C (Fig. 4c) expression was significantly higher in cell line models of basal-squamous disease when compared with luminal and “non-type” cell lines. (TFAP2A: *p* = 0.0095, TFAP2C: *p* = 0.0095; Mann–Whitney U test). In addition, increased levels of TFAP2A expression remained statistically significant even after removing HT1197 cells from the analysis (*p* = 0.0238; Mann–Whitney U test).This was additionally confirmed by western blotting showing that while expression of both PPARɣ isoforms (ɣ1 and ɣ2) was variable across BC cell lines, the basal-squamous cell lines SCaBER, 5637, HT1376, and HT1197 expressed relatively high TFAP2A and TFAP2C expression (Fig. 4d) in comparison to luminal and “non-type” lines.

**Fig. 5:**
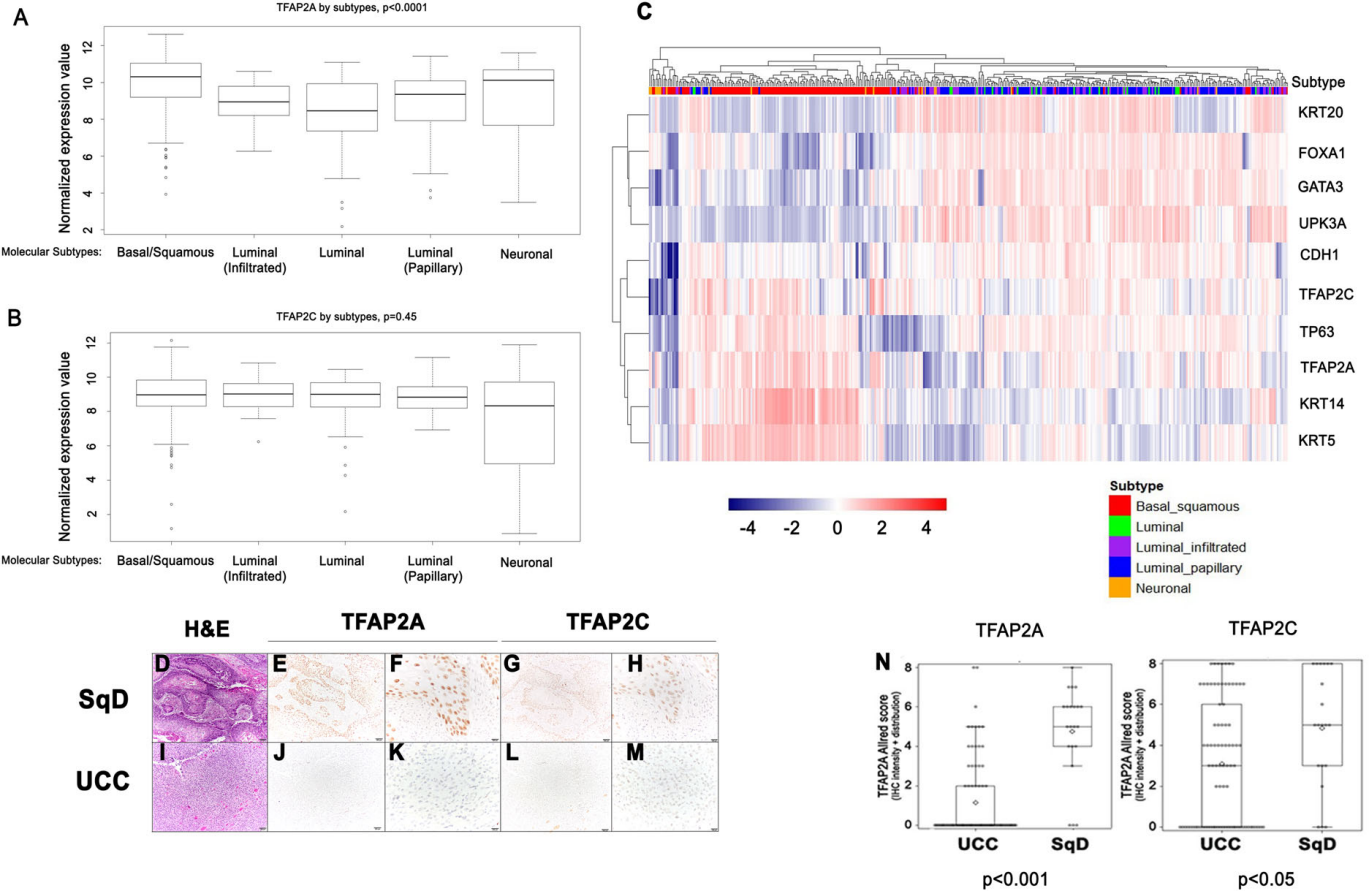
TFAP2A and TFAP2C expression is associated with basal-squamous human bladder cancer. **a**, **b** Relationship between molecular subtype and expression of TFAP2A and TFAP2C was examined using data compiled through the TCGA bladder cancer study. While TFAP2A (**a**; Kruskal–Wallis H test; *p* < 0.0001) expression was significantly elevated in the basal-squamous molecular subtype, TFAP2C (**b**) expression was not significantly associated with any subtype at the mRNA level. **c** Hierarchical clustering analysis using data compiled through the same TCGA bladder cancer study shows tumors that express TFAP2A and TFAP2C cluster with tumors expressing additional markers of basal bladder cancer. **d–m** Representative images of H&E (**d**, **i**) and IHC staining for TFAP2A (**e**, **f**, **j**, and **k**), TFAP2C (**g**, **h**, **l**, and **m**) from human BC specimens (**d**–**h**: SqD, UCC: **i**–**m**). **n** TFAP2A (*p* < 0.001; Wilcoxon rank sum) and TFAP2C (*p* < 0.05; Wilcoxon rank sum) protein expression are significantly higher in SqD compared with UCC.

Next, we analyzed publically available data from The Cancer Genome Atlas (TCGA) BC study, as well as our own in-house tumor cohort. Computational analysis of TCGA data showed TFAP2A mRNA expression is significantly enriched in basal-squamous tumors relative to other subtypes (Fig. 5a; *p* < 0.0001). While TFAP2C mRNA levels were not significantly correlated with molecular subtype (Fig. 5b; *p* = 0.45), high expression of both TFAP2A and TFAP2C clustered with basal-squamous markers including KRT5, KRT14, and TP63 (Fig. 5c). Furthermore, while TFAP2A and TFAP2C overexpression were significantly correlated with SqD in the TCGA cohort (*p* = 8.28E−10 and *p* = 0.00457, respectively; Wilcoxon Rank sum; Supplementary Fig. S6), neither was associated with tumor stage, presence of lymph node metastases, lymphovascular invasion or vital status in the TCGA cohort (Supplementary Fig. S6). Immunohistochemistry performed on our previously described^22^, in-house cohort of over 100 BC patients confirmed areas of invasive carcinoma with SqD expressed higher levels of TFAP2A and TFAP2C relative to invasive conventional UCC (Fig. 5d–n and Supplementary Table S2; *p* < 0.001, *p* = 0.015, respectively, Wilcoxon rank sum test). In addition, analysis of our BC cohort (both UCC and SqD), we show TFAP2A expression at the protein level is significantly associated with the presence of lymph node metastasis (Supplementary Table S3: TFAP2A: *p* = 0.049 TFAP2C: *p* = 0.635; Chi-square test). Moreover, increased TFAP2C protein expression was associated with distant recurrence (TFAP2A: *p* = 0.137, TFAP2C: *p* = 0.037; Chi-square test; Supplementary Table S3). These observations identify TFAP2A and TFAP2C as markers of the aggressive basal-squamous molecular subtype of human BC.

### TFAP2A is positively correlated with TP63 in human BC samples and also positively regulates TP63 expression in human BC cell

**Fig. 6:**
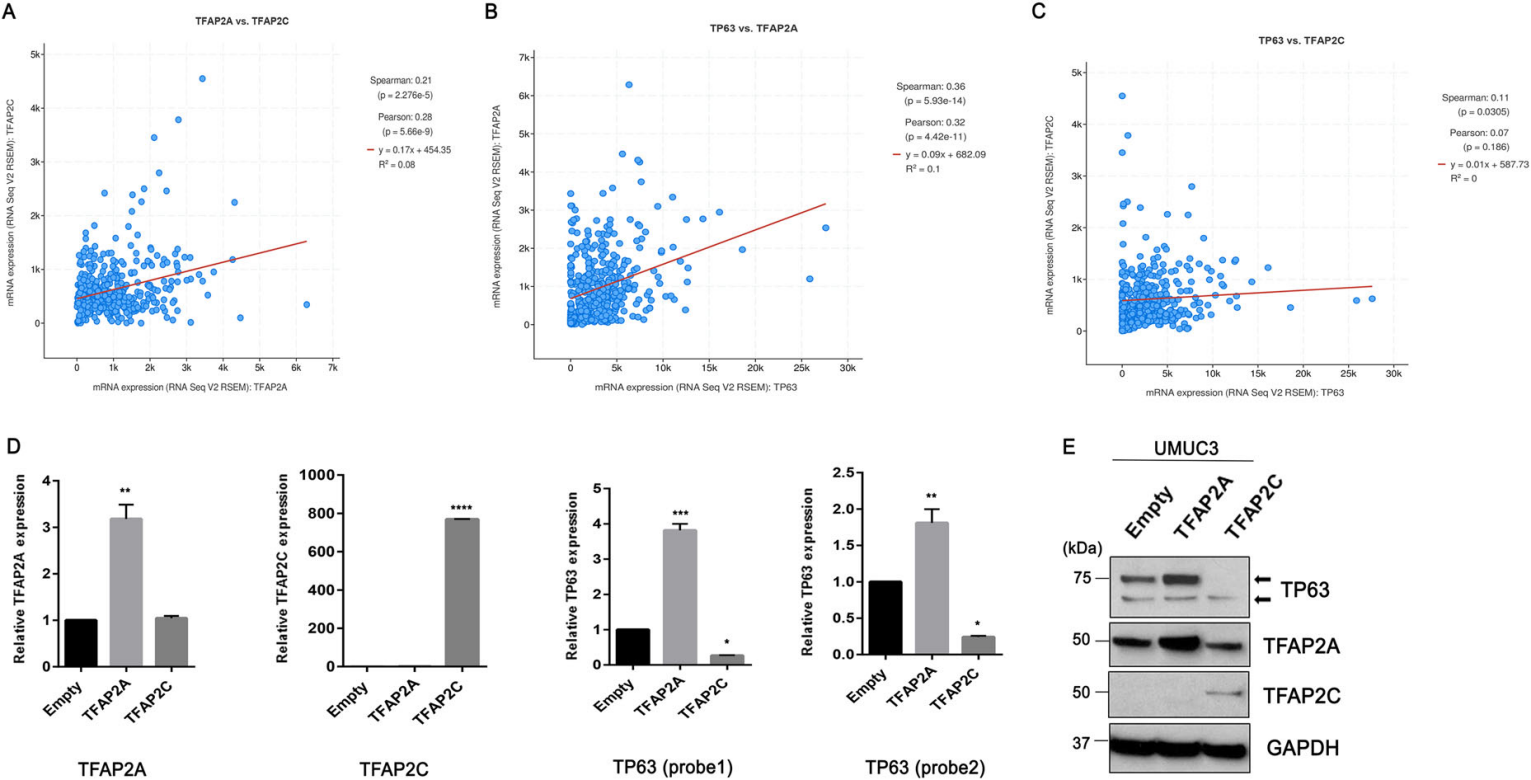
TFAP2A is positively correlated with TP63 in human bladder cancer samples and also positively regulates TP63 expression in human bladder cancer cell. **a–c** Spearman’s rank correlation analysis between TFAP2A and TFAP2C (**a**), TFAP2A and TP63 (**b**), TFAP2C and TP63 (**c**) mRNA in human bladder urothelial carcinoma. **d** q-RT-PCR of TP63, TFAP2A, TFAP2C in TFAP2A or TFAP2C overexpresssing UMUC3 cells. Probe1 and 2 for TP63 were used to detect the boundary of exon 1–2 (full length) and exon 4–5, 6–7 (both full length and variants), respectively. Data are expressed as the mean ± S.D. from twice independent experiments. *p < 0.05, **p < 0.01, ***p < 0.001, ****p < 0.0001, one-way ANOVA with post hoc multiple comparison (Dunnett). **e** Western blot analysis of TP63, TFAP2A, TFAP2C in TFAP2A or TFAP2C overexpresssing UMUC3 cells. Arrow shows full length (~75 kDa) and variants, respectively.

Expression of the transcripton factor TP63 plays an important role in the basal-squamous subtype of BC^23,24^. Therefore, we next set out to determine if any relationship between TFAP2A/TFAP2C and TP63 could be identified. Analysis of publically available human data from the TCGA study^25^ shows TFAP2A expression is significantly positively correlated with TFAP2C (Spearman’s rank correlation; *r* = 0.21, *p* = 2.276E−5, Fig. 6a). In addition, TP63 expression is also positively correlated with TFAP2A expression (Spearman’s rank correlation; *r* = 0.36, *p* = 5.93E−14; Fig. 6b) and (although weaker), with TFAP2C (Spearman’s rank correlation; *r* = 0.11, *p* = 0.0305, Fig. 6c). Based on these observations, we next investigate the effect of overexpressing TFAP2A and TFAP2C on TP63 expression in a human BC cell line. Overexpression of TFAP2A significantly increased expression of TP63 at the mRNA (Fig. 6d) and protein (Fig. 6e) levels. Suprisingly, TFAP2C overexpression appeared to have the opposite effect, resulting in reduced TP63 expression levels at the mRNA (Fig. 6d) and protein (Fig. 6e) levels. Interestingly, TAp63 (75KDa) appeared to be most robustly influenced by these experiments. While further work is required, these results suggest an potential regulatory relationship between these transcription factors.

### TFAP2A and TFAP2C expression control the aggressive phenotype associated with basal-squamous bladder cancer cells

**Fig. 7:**
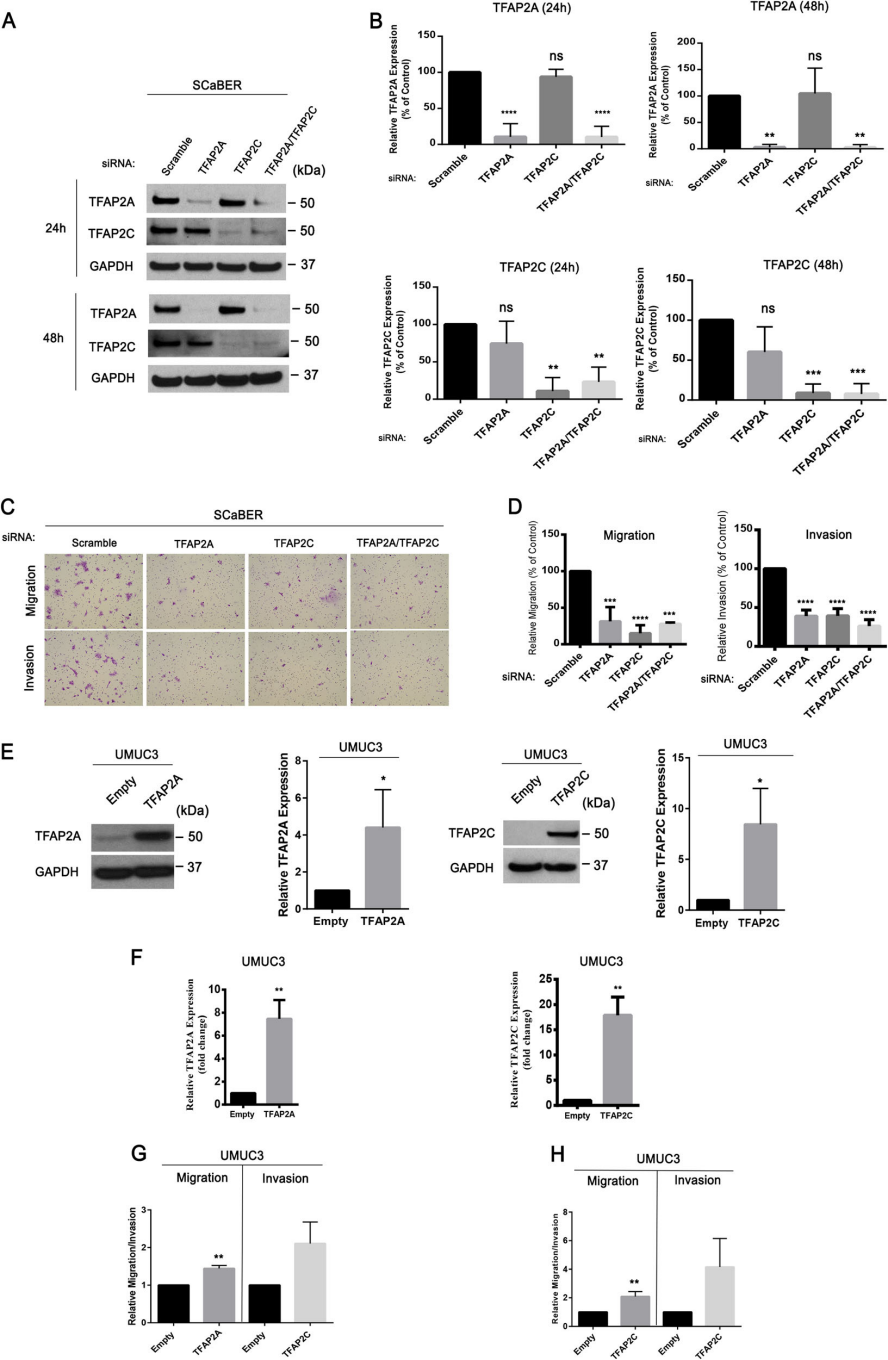
Expression of TFAP2A and TFAP2C influences BC cell migration and invasion. **a** Western blotting analysis of TFAP2A and TFAP2C expression in SCaBER cells after siRNA transfection. SiRNAs included Scrambled (negative control), as well as constructs targeting TFAP2A and TFAP2C individually and in combination. **b** Densitometric analysis of western blotting results for TFAP2A and TFAP2C expression for data depicted in (**a**). TFAP2A and TFAP2C expression was normalized to GAPDH. Data are expressed as the mean ± S.D from three independent experiments. **p < 0.01, ***p < 0.001, ****p < 0.0001, one-way ANOVA with post hoc multiple comparison (Dunnett). **c** Representative images following migration and Invasion assays using SCaBER cells transfected with siRNA for Scrambled construct, TFAP2A, TFAP2C, and TFAP2A/TFAP2C. **d** Relative migration and invasion of SCaBER cells transfected with siRNA. Data are expressed as the mean ± S.D. from three independent experiments. ***p < 0.001, ****p < 0.0001, one-way ANOVA with post hoc multiple comparison (Dunnett). **e** Western blotting analysis of TFAP2A and TFAP2C protein expression levels in UMUC3 cells overexpressing TFAP2A (left) or TFAP2C (right). Also included is densitometric analysis of western blotting data for TFAP2A and TFAP2C. **f** q-RT-PCR analysis of TFAP2A and TFAP2C expression in UMUC3 cells overexpressing TFAP2A and TFAP2C. **g** Migration and Invasion assay of UMUC3 stable cells overexpressing TFAP2A. **h** Migration and Invasion assay of UMUC3 cell overexpressing TFAP2C. Data are expressed as the mean ± S.D. from three independent experiments. **p < 0.01 (Student’s *t* test).

Because of the close association between TFAP2A and TFAP2C expression and the aggressive basal-squamous subtype, as well as their association with lymph node metastasis and distant recurrence in our tumor cohort, we hypothesized these transcriptional regulators may promote disease aggressiveness. SCaBER cells express high levels of TFAP2A and TFAP2C, and are suitable for transient transfection experiments. Therefore, SCaBER was used to study the effect of TFAP2A and/or TFAP2C knockdown on migration and invasion as well as proliferation. Western blotting (Fig. 7a, b) showed we successfully decreased expression of TFAP2A and TFAP2C at 24 and 48 h post transfection. In vitro migration and invasion assays show that individual knockdown of TFAP2A or TFAP2C, as well as combined knockdown of TFAP2A and TFAP2C results in significantly reduced migration and invasion of SCaBER cells compared with knockdown of scramble siRNA control (Fig. 7c, d). In addition, individual or combined knockdown of TFAP2A and TFAP2C had no effect on cell proliferation (Supplementary Fig. S7). UMUC3 cells are easily transfectable, express relatively low levels of TFAP2A, and no detectable TFAP2C (see Fig. 4d). Therefore, we additionally established UMUC3 cells stably overexpressing TFAP2A (UMUC3-TFAP2A) and TFAP2C (UMUC3-TFAP2C). Western blotting and Q-RT-PCR confirmed stable overexpression of TFAP2A and TFAP2C in UMUC3 at the protein and mRNA levels, respectively (Fig. 7e, f). Migration and invasion assays using these stable cells showed that increased TFAP2A or TFAP2C in UMUC3 cells significantly enhanced migration and invasion (Fig. 7g, h). Thus, these results suggest TFAP2A and TFAP2C is important for the control phenotypic aggressiveness associated with basal-squamous BC.

### Overexpression of TFAP2A or TFAP2C in bladder cancer cells promotes tumorigenicity following tissue recombination xenografting

**Fig. 8:**
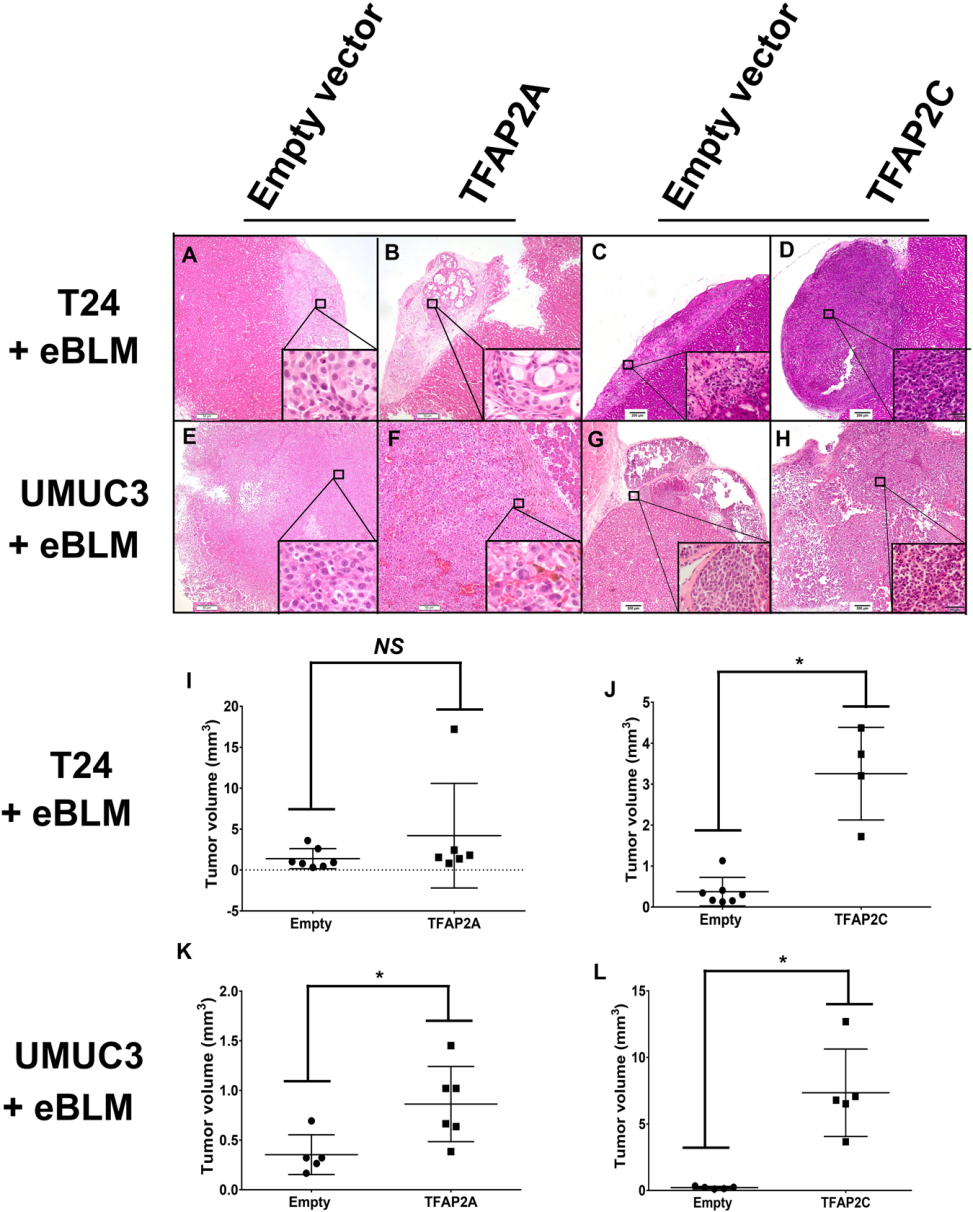
Overexpression of TFAP2A or TFAP2C in bladder cancer cell promotes tumorigenicity in tissue recombination xenografting assays. Following genetic manipulation and recombination with embryonic rat bladder mesenchyme, tissue recombinants were inserted underneath the renal capsule as described in materials and methods. **a–d** Hematoxylin and eosin staining of T24 cells engineered to stably express empty vector (**a**) or TFAP2A (**b**), empty vector (**c**) or TFAP2C (**d**). **e–h** Hematoxylin and eosin staining of UMUC3 cells engineered to stably express empty vector (**e**) or TFAP2A (**f**), empty vector (**g**) or TFAP2C (**h**). Overexpression of TFAP2A in T24 had no significant effect on tumor volume in the tissue recombination assay (**i**), while overexpression of TFAP2C significantly increased tumor volume of T24 recombinants (**j**). Overexpression of TFAP2A (**k**) and TFAP2C (**l**) significantly increased tumor volume of UMUC3 recombinants. Data are expressed as the medians ± S.D. *p < 0.05, **p < 0.01, ns: not significant, Mann–Whitney U test.

We next utilized the tissue recombination system to investigate the impact of TFAP2A and TFAP2C overexpression on tumorigenicity and the ability to drive morphologic changes, such as SqD. For these experiments, we utilized UMUC3 cells stably overexpressing TFAP2A or TFAP2C (see Fig. 7e), as well as T24 cells stably overexpressing TFAP2A or TFAP2C (see Supplementary Fig. S8). These cells were chosen because of their relatively low expression of TFAP2A and TFAP2C (see Fig. 4). These cell lines and empty vector control cells were recombined with embryonic bladder mesenchyme, and inserted under the renal capsule of immunocompromised mice as previously described^11^ and indicated in the Materials and methods section. Two months following implantation, T24-EV xenografts were weakly tumorigenic and tumor volume was not significantly increased following TFAP2A overexpression (Fig. 8a, b; *p* = 0.2343; quantified in 8i). However, T24 tumor xenograft volume was significantly increased following expression of TFAP2C (Fig. 8c, d; *p* < 0.01; quantified in 8j) 2 months following implantation. In addition, UMUC3-associated tumor xenograft volume was significantly increased following overexpression of TFAP2A (Fig. 8e, f; *p* < 0.05; quantified in 8k) and TFAP2C (Fig. 8g, h; *p* < 0.01; quantified in 8l). However, we failed to detect SqD in any of our tissue recombination xenograft experiments. These observations suggests that TFAP2C, and to a lesser extent, TFAP2A promote tumorigenicity within the tissue recombination xenografting system, but not sufficient to drive SqD.

## Discussion

**Fig. 9:**
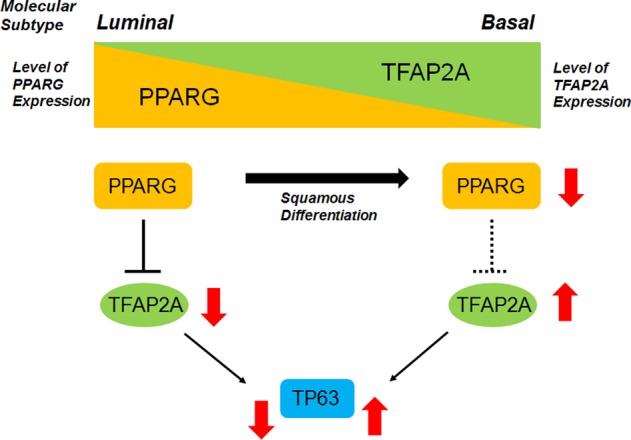
Schematic diagram of hypothetical model in this study. During BC progression, squamous differentiation (SqD) was often obsearved in advanced bladder cancer. Molecular basal subtypes is relatively associated with SqD than luminal subtypes. In luminal subtypes, TFAP2A expression is repressed by PPARɣ receptor. On the other hand, TFAP2A is expressed in basal subtypes due to the loss or malfunction of PPARɣ in terms of the capabilities to repress TFAP2A. TFAP2A upregulates TP63 expression and cooperates to induce SqD with TP63 as well as other factors in basal subtypes of human BC.

Based on previous work suggesting PPARɣ activity is critical for maintaining a luminal gene expression program^9,11^, we undertook a pharmacologic screen to identify PPARɣ-repressed transcription factors that act as potential master regulators of SqD and/or the basal-squamous molecular subtype (Fig. 1). This screen identified TFAP2A as one such PPARɣ-repressed TF that is overexpressed in human basal-squamous disease. Collectively, our results suggest that while TFAP2A expression is normally repressed by PPARɣ in normal urothelium and luminal subtypes of BC, inactivation of PPARɣ via as of yet unidentified mechansims results in increased TFAP2A expression. As our data fail to show the ability of TFAP2A (or TFAP2C) to directly drive SqD, we hypothesize that TFAP2 family members either cooperate with other factors to drive these phenotypes and/or expression of TFAP2 family members maintain a basal-squamous state, enabling phenotypic stability. Whatever the mechanism, increased TFAP2A then results to increase the expression of transcription factors important for SqD, including TP63 (Fig. 9), and potentially TFAP2C. While other factors cooperarte with TFAP2A and further studies are required, this is the model we propose regarding the contribution of TFAP2A to the basal-squamous fate of BC.

TFAP2A belongs to the TFAP2 family, which consists of five members (TFAP2A, 2B, 2C, 2D, 2E). In addition to TFAP2 family members being essential for neural crest development^26^ and estrogen receptor binding and subsequent long-range chromatin interactions in breast cells^27^, both TFAP2A and TFAP2C are implicated in keratinocyte differentiation^21,28,29^ and squamous cancers^30^ independent of anatomic site. At the molecular level in normal keratinocytes, TP63 (itself implicated in the basal subtype of BC^24^) activates TFAP2C expression to promote normal skin differentiation^19,31^, and cooperates with TFAP2A and TFAP2C to regulate TP63 target gene expression^20^. Also, *Tfap2a*, and *Tfap2c* knockout in mice produces pathologic skin disease^29^, potentially by impacting epidermal growth factor receptor signaling which is also implicated in basal BC. Our study showed that TFAP2A overexpression acts to increase TP63 expression in a human BC cell line. While more work is required, it is interesting to postulate that PPARɣ may impact TP63 levels, potentially by controlling TFAP2A expression. However, individual TFAP2A or TFAP2C overexpression in UMUC3 and T24 cells did not induce SqD. This observation suggests that TFAP2A and/or TFAP2C expression may require additional transcription factors other than TP63 to induce SqD. In addition to TP63, other cellular circuitry also may be implicated in the development of SqD (reviewed in ref. ^32^). Because the detailed mechanisms remains to be unclear, further research is required to investigate the mechanisms behind these events in the future.

Ligand-dependent increases in the expression of PPARɣ target genes involves release of corepressor complexes including nuclear receptor corepressor 1 (NCoR1) and 2 (NCoR2/SMRT) and other factors including histone deacetylases (reviewed in ref. ^33^). This process results in the recruitment of general transcription machinery and increased gene expression. However, the mechanisms of ligand-dependent repression of PPARɣ target genes are less clear. The fact that TFAP2A is repressed by PPARɣ activation in UMUC1, SW780, and 5637, and our observation that co-treatment with a PPARɣ antagonists abrogates TZD-induced TFAP2A regulation in all three models suggests the existence of a general and shared mechanism. Although the exact mechanism remains to be identified, our experiments using both a PPARɣ antagonist and siRNA support a requirement for a functional PPARɣ for the repression of TFAP2A. While PPARɣ and other PPARs can directly interfere with the ability of other transcription factors to regulate gene expression via a process referred to as transrepression^33^, it is not clear if this mechanism is responsible for ligand-dependent repression of TFAP2A by PPARɣ. As PPARɣ plays an important role in the BC cell autonomous regulation of cytokines and response to immunotherapy^8,34^, further studies are required in the future.

We additionally report here that both TFAP2A and TFAP2C are expressed at high levels in basal-squamous BC, as well as in areas of SqD (Fig. 5). Indeed, the association between increased TFAP2A and TFAP2C expression was observed in both the TCGA BC cohort, as well as our in-house tumor cohort. Importantly, the fact that TFAP2A and TFAP2C promote in vitro surrogate measures of aggressive behavior typically associated with basal-squamous BC (Fig. 7), as well as in vivo tumorigenesis is in agreement with their association with lymph node metastasis and distant recurrence in our cohort, and further suggests an important role for these factors in BC. However, the fact that expression of TFAP2A and TFAP2C were not associated with poor prognoistic indicators or outcome in the TCGA suggest additonal efforts are needed to resolve this discrepancy. In addition to the fact that SqD and the basal-squamous subtype has been associated with enhanced overall survival following neoadjuvant chemotherapy and cystectomy^35^, TFAP2A was previously identified as an independent predictor of good response to cisplatin treatment in BC patients^36^. While these observations further link TFAP2 family member expression to variant morphology in BC, we did not detect SqD in our tissue recombinants. However, this is not surprising as TFAP2 family members undoubtedly require the action of additional combinatorial factors^37,38^ to mediate a squamous cell fate in vivo. Taken together, this study implicates TFAP2A and TFAP2C in the Basal molecular subtype of BC, as well as associated SqD.

## Materials and methods

### Cell culture

All BC cell lines were purchased as described previously and authentiticity was confirmed by short tandem repeat (STR) analysis^11^. The Lenti-X 293T cell line (Takara, Mountain View, CA) was maintained in Dulbecco’s modified Eagle’s medium (DMEM) with high glucose, L-glutamine with 10% Tet System Approved FBS (Takara, Mountain View, CA).

### PPARɣ agonist and antagonist treatment

One day before transfection, 5637, UMUC1, SW780 BC cells were plated (4 × 10^5^ cells/well) in 6-well plates (Corning Inc., Corning, NY) in complete culture medium containing 10% FBS and allowed to attach overnight. On the following day, culture medium were replaced with serum-free medium and incubated for 24 h. After 24 h, medium was replaced with serum-free medium containing either Dimethyl sulfoxide (DMSO; Sigma, St. Louis, MO) vehicle control or rosiglitazone (TZD; 1 μM; TOCRIS, Bristol, UK) and incubated for 48 h. RNA and protein were harvested as described below in the pertinent methods sections. For experiments utilizing the PPARɣ antagonist GW9662 (TOCRIS, Bristol, UK), cells were pretreated with GW9662 at a final concentration of 1 or 5 μM for 1 h before the addition of TZD.

### RNA-sequencing and computational analysis

cDNA libraries were prepared using the NEXTflex™ Illumina Rapid Directional RNA-Seq Library Prep Kit (Bio Scientific) as per the manufacturer’s instructions. Denatured libraries were diluted to 10 pM by pre-chilled hybridization buffer and loaded onto a TruSeq SR v3 flow cells on an Illumina HiSeq 2500 for 50 cycles using a single-read recipe (TrueSeq SBS Kit v3) and run for 50 cycles using a single-read recipe according to the manufacturer’s instructions. De-multiplexed and adapter-trimmed sequencing reads were generated using Illumina bcl2fastq (released version 2.18.0.12) allowing no mismatches in the index read. The sequencing reads were subjected to quality filtering used FASTX-Toolkit (http://hannonlab.cshl.edu/fastx_toolkit) to keep only reads that have at least 80% of bases with a quality score of 20 or more (conducted by fastq_quality_filter function) and reads left with >10 bases after being trimmed with reads with a quality score of <20 (conducted by fastq_quality_trimmer function). Filtered reads were mapped to the human reference genome (GRCh38) using TopHat (version 2.0.9)^39^ supplied by Ensembl annotation file; GRCh38.78.gtf. After normalization was performed via the median of the geometric means of fragment counts across all libraries, differential expression was determined using the Cuffdiff tool which is available in Cufflinks version 2.2.1^40^. All genes passing FDR criteria of 0.05 were considered differentially expressed genes. Venn diagrams and heatmaps were generated using limma in R. GO analysis was performed using DAVID Bioinformatics Resources 6.8 (https://david.ncifcrf.gov). For computational analysis of TCGA data for TFAP2A and TFAP2C expression in Fig. 5, RNA-seq expression data was obtained from the Genomic Data Commons (https://portal.gdc.cancer.gov/). Expression was log2 normalized and clustered with the genes listed using the heatmap package in R. pheatmap: Pretty Heatmaps. R package version 1.0.10 (https://CRAN.R-project.org/package = pheatmap) and correlation distance. Annotation data including expression subtype were obtained from the supplemental data available with the TCGA bladder cancer project. Gene expression data for the TCGA BLCA cohort in Suppelementary Fig. S3 was downloaded from the Broad Institute’s Firehose GDAC (https://gdac.broadinstitute.org/), and TFAP2A and TFAP2C expression was quantified as log2(RSEM + 1). The Wilcoxon rank sum test was applied to compare TFAP2A and TFAP2C expression levels in subjects with and without squamous differentiation based on clinical data from ref. ^10^. R 3.5.0 (R Core Team) (https://www.r-project.org) was used to perform statistical analyses and create Supplementary Fig. S3.

### RNA extraction and quantitative real-time PCR (q-RT-PCR)

Total RNA was extracted using the RNeasy approach (Qiagen, Hilden, Germany) according to manufacturer protocol. For cDNA synthesis, reverse transcription was performed using M-MLV reverse transcriptase (Thermo Fisher) via manufacturer instructions. q-RT-PCR was performed using QuantaStudio7 Real-Time PCR System (Applied Biosystems, Foster City, CA). Taqman probes used in this study were as follows. TFAP2A (Hs01029413_m1), TFAP2C (Hs00231476_m1), FABP4 (Hs01086177_m1), PPARG (Hs00234592_m1), TP63 (Hs00978339 and Hs00978343). Relative gene expression change was calculated by deltadeltaCt method. 18S ribosomal RNA was used as an endogenous reference.

### Western blotting

Western blotting was performed as described previously^11^. Primary antibodies were used as shown in Supplementary Table S4.

### Plasmid construction

For construction of pLVX-IRES-TFAP2A plasmid, cDNA was amplified by Accuprime Super Mix (Invitrogen, Carlsbad, CA) using Ultimate ORF TFAP2A/AP2alpha (IOH46467, Thermo Fisher, Waltham, MA) as a template and primers including forward (5′-TCTGAATTCACCATGCTTTGGAAATTGACGGATAATATC-3′), reverse (5′-GCTCGAGTTAAACCTTATCGTCGTCATCCTTGTAATCCAGCTTTCTGTGCTTCTCCTCTTTGTCACTGCTTTTG-3′). PCR products were digested with EcoRI and XhoI (New England Biolabs; Ipswich, MA) and ligated into the pLVX-IRES-Neo^R^ plasmid. pLenti6.3/V5-TFAP2C was generated by mixing 1 μl of Ultimate ORF TFAP2C (IOH28749, Thermo Fisher, Waltham, MA) and 1 μl of pLenti6.3/V5-DEST (Thermo Fisher, Waltham, MA) in TE buffer (pH 8.0) and incubating with 2 μl of LR Clonase^TM^ II at 25 °C for 1 h. This mixture was subsequently incubated with 1 μl of ProteinaseK (Sigma, St. Louis, MO) and transformed into chemically competent *E. coli* cells. To construct pLVX-EF1alpha-IRES-TFAP2C plasmid, PCR was performed by Platinum Taq polymerase (Thermo Fisher, Waltham, MA) based on pLenti6.3/V5-TFAP2C plasmid as a template. Primers were used Forward (5′-TTTACTAGTATGTTGTGGAAAATAACCGATAATGTC-3′) and Reverse (5′-TTAGGATCCCTAACCGGTACGCGTAGAATCGAG-3′). The amplified PCR product was digested with SpeI and BamHI and was ligated into pLVX-EF1alpha-IRES-puro^r^ (Takara, Mountain View, CA).

### Generation of stable cell lines

pLVX-IRES-TFAP2A or pLVX-EF1alpha-IRES-TFAP2C plasmid were transfected in Lenti-X 293T cells with Lenti-X Packaging Single Shots (VSV-G) (Takara, Mountain View, CA). After 48 h, medium was collected and virus titer was measured by Lenti-X GoStix^TM^ (Takara, Mountain View, CA). Collected medium containing Lentivirus was filtered through a 0.45 μm polyethersulfone membrane (GE Healthcare, Chicago, IL). For lentivirus infection, T24 or UMUC3 cells were seeded onto 6-well plates to reach ~70% confluency. Medium was replaced with 2 ml of virus medium as prepared above with polybrene. After 48 h incubation, medium was removed and replaced with medium containing 2 mg/ml G418 (for pLVX-IRES-TFAP2A) or 1 μg/ml puromycin (for pLVX-EF1alpha-IRES-TFAP2C).

### siRNA transfection

siRNA transfection was performed using a reverse transfection method with lipofectamine 3000 (Thermo Fisher, Waltham, MA). Non-targeting (Scrambled; D-001810-01-05), TFAP2A (L-006348-02-0005), TFAP2C (L-005238-00-0005), and PPARG (L-003436-00) siRNAs were obtained from Dharmacon (Lafayette, CO). siRNA and lipofectamine 3000 were added to separate aliquots of OPTi-MEM medium, respectively, and incubated for 5 min. Subsequently, siRNA and lipofectamine 3000 in OPTi-MEM were mixed and incubated for 20 min. After incubation, siRNA-lipofectamine 3000 complex was added to 2 ml cell suspension containing 4 × 10^5^ target cells per well in a 6-well plate.

### Tumor cohort

Of the 309 consecutive cases used to create the previously described TMA^22^, 270 had invasive carcinoma. See ref. ^22^ for detailed cohort information. Of these 270 carcinomas, 104 cases had evaluable tissue after IHC to determine the relationship between TFAP2A and/or TFAP2C expression and tumor morphology. In addition, 94 and 96 cases had evaluable tissue after IHC to determine if their existed any correlation between the presence of lymph node metastases and TFAP2A or TFAP2C expression, respectively. Ninety seven and 99 cases had evaluable tissue after IHC to identify the existence of a correlation between distant recurrence and TFAP2A or TFAP2C expression, respectively.

### Immunohistochemistry of human bladder cancer tissue

All human studies were performed in accordance with approved protocols from the Institutional Review Board of Pennsylvania State University. Immunohistochemistry (IHC) on our previously described human BC TMA^22^ was performed via established methods^41^. Primary antibodies are referenced in Supplementary Table S4. Nuclear expression was quantified by Allred score, which combines a measure of expression intensity (range 0–3, no expression to highest expression) to a measure of expression area (range 0–5, no expression to diffuse expression) to give a score ranging form 0 to 8^42^.

### Migration and invasion assays

Falcon 8.0 μm transparent PET membrane cell culture inserts were used for migration assays and Matrigel Transwell inserts (Corning Inc, Corning, NY) was used for invasion assays. Briefly, 750 μl of medium containing 10% FBS was added to the space between cell culture insert or a Matrigel Transwell insert (Corning Inc, Corning, NY)) and the well within a 24-well plate. A total of 1 × 10^5^ cells were suspended in 500 μl of serum-free medium and added to the transwell insert. T24 and UMUC3 cells were incubated for 12 h (migration), or 24 h (invasion). SCaBER cells were incubated for 12 h (migration) or 15 h (invasion). After incubation, migrated and invaded cells were stained with 0.5% crystal violet (Sigma) in 20% methanol (Sigma, St. Louis, MO) for 20 min, and residual cells that had not moved through the transwell were removed by gentle swabbing with a Q-tip (Unilever, Trumbull, CT). Cell numbers were counted in microscopic field (×100 magnification). All experiments were repeated three times in triplicate.

### Tissue recombination xenografting

All animal experiments were performed in accordance with approved protocols from the Institutional Animal Care and Use Committee of Pennsylvania State University. Pregnant rats (Harlan Laboratories, Tama, FL) were sacrificed at embryonic day 16 (plug day = 0). Isolation of embryonic bladder mesenchyme (eBLM), preparation of tissue recombinants, and kidney capsule surgeries were performed as described previously^7,11,43^. UMUC3 and T24 (1 × 10^5^) cells overexpressing TFAP2A or TFAP2C were used for each graft. Recombinants were surgically implanted under the kidney capsule of seven 2-month-old male SCID mice (Jackson Laboratoires; Bar Harbor, ME). Four weeks following implantation, mice bearing UMUC3 cells were sacrificed, whereas mice bearing T24 cells were sacrificed 8 weeks after surgery, respectively. Tumor radius was measured through cellSens software on an Olympus CX41 microscope, and tumor volumes were approximated by the calculation tumor volume, *V* (mm^3^) = π/6 × *W* × *H* × *L* (*V*: volume, *W*: width, *H*: height, *L*: length). Individuals responsible for analysis of recombinants were blinded as to whether tissue was isolated from control or experimental animals.

### Statistical analysis

Statistical analysis was performed using SAS (SAS Institute Incoprotated, Cary, NC) and GraphPad Prism6 (GraphPad Software, San Diego, CA). Sample sizes for in vivo studies were calculated based on prior studies^7,11,43,44^. Randomization was deemed not to be necessary for these studies. Normally distributed In vitro data were analyzed using parametric tests (Student’s *t* test and analysis of variance (ANOVA) with Tukey’s and Dunnett’s post hoc correction for multiple comparison), while in vivo and clinical data was analyzed using non-parametric (Wilcoxon rank sum test and Kruskal–Wallis H test) approaches. Chi-square tests were used to test for associations between TFAP2A and TFAP2C expression with lymph node metastasis and distant recurrence. In vitro results are expressed as the mean ± S.D. from three independent experiments. *p* < 0.05 was considered as a statistically significant.

## Supplementary information

STableS1

STableS2

STableS3

STableS4

FigS1

FigS2

FigS3

FigS4

FigS5

FigS6

FigS7

FigS8
