# Supplementary material for: Repression of transcription factor AP-2 alpha by PPARγ reveals a novel transcriptional circuit in basal-squamous bladder cancer
Source: Oncogenesis. 2019 Nov 26;8(12):69. doi: 10.1038/s41389-019-0178-3 (PMC6879593; doi:10.1038/s41389-019-0178-3)
Supplement: Supplementary file 1 — STableS1 [file 41389_2019_178_MOESM1_ESM.docx]

**Supplementary Table S1:**

**Significantly upregulated and downregulated genes following rosiglitazone treatment of UMUC1, SW780 and 5637 bladder cancer cells (FDR-adjusted p <0.05)**

1)Genes uniquely upregulated in UMUC1 cells following rosiglitazone treatment

2)Genes uniquely upregulated in SW780 cells following rosiglitazone treatment

3)Genes uniquely upregulated in 5637 cells following rosiglitazone treatment

4)Genes upregulated in common in both UMUC1 and SW780 cells following rosiglitazone treatment

5)Genes upregulated in common in both SW780 and 5637 cells following rosiglitazone treatment

6)Genes upregulated in common in both UMUC1 and 5637 cells following rosiglitazone treatment

7)Genes upregulated in common in UMUC1, SW780, and 5637 cells following rosiglitazone treatment

8)Genes uniquely downregulated in UMUC1 cells following rosiglitazone treatment

9)Genes uniquely downregulated in SW780 cells following rosiglitazone treatment

10)Genes uniquely downregulated in 5637 cells following rosiglitazone treatment

11)Genes downregulated in common in UMUC1 and SW780 cells following rosiglitazone treatment

12)Genes downregulated in common in SW780 and 5637 cells following rosiglitazone treatment

13)Genes downregulated in common in UMUC1 and 5637 cells following rosiglitazone treatment

14)Genes downregulated in common in UMUC1, SW780, and 5637 cells following rosiglitazone treatment

1) Genes uniquely upregulated in UMUC1 cells following rosiglitazone treatment

Genes uniquely upregulated in UMUC1 cells following rosiglitazone treatment (continued)

Genes uniquely upregulated in UMUC1 cells following rosiglitazone treatment (continued)

Genes uniquely upregulated in UMUC1 cells following rosiglitazone treatment (continued)

Genes uniquely upregulated in UMUC1 cells following rosiglitazone treatment (continued)

Genes uniquely upregulated in UMUC1 cells following rosiglitazone treatment (continued)

Genes uniquely upregulated in UMUC1 cells following rosiglitazone treatment (continued)

2) Genes uniquely upregulated in SW780 cells following rosiglitazone treatment

3) Genes uniquely upregulated in 5637 cells following rosiglitazone treatment

4)Genes upregulated in common in both UMUC1 and SW780 cells following rosiglitazone treatment

5)Genes upregulated in common in both SW780 and 5637 cells following rosiglitazone treatment

6)Genes upregulated in common in both UMUC1 and 5637 cells following rosiglitazone treatment

7)Genes upregulated in common in UMUC1, SW780, and 5637 cells following rosiglitazone treatment

8)Genes uniquely downregulated in UMUC1 cells following rosiglitazone treatment

Genes uniquely downregulated in UMUC1 cells following rosiglitazone treatment (continued)

Genes uniquely downregulated in UMUC1 cells following rosiglitazone treatment (continued)

Genes uniquely downregulated in UMUC1 cells following rosiglitazone treatment (continued)

Genes uniquely downregulated in UMUC1 cells following rosiglitazone treatment (continued)

Genes uniquely downregulated in UMUC1 cells following rosiglitazone treatment (continued)

Genes uniquely downregulated in UMUC1 cells following rosiglitazone treatment (continued)

Genes uniquely downregulated in UMUC1 cells following rosiglitazone treatment (continued)

9)Genes uniquely downregulated in SW780 cells following rosiglitazone treatment

10)Genes uniquely downregulated in 5637 cells following rosiglitazone treatment

11)Genes downregulated in common in UMUC1 and SW780 cells following rosiglitazone treatment

12)Genes downregulated in common in SW780 and 5637 cells following rosiglitazone treatment

13)Genes downregulated in common in UMUC1 and 5637 cells following rosiglitazone treatment

14)Genes downregulated in common in UMUC1, SW780, and 5637 cells following rosiglitazone treatment
