# Supplementary material for: Repression of transcription factor AP-2 alpha by PPARγ reveals a novel transcriptional circuit in basal-squamous bladder cancer
Source: Oncogenesis. 2019 Nov 26;8(12):69. doi: 10.1038/s41389-019-0178-3 (PMC6879593; doi:10.1038/s41389-019-0178-3)
Supplement: Supplementary file 2 — STableS2 [file 41389_2019_178_MOESM2_ESM.docx]

| **Supplementary Table S2: TFAP2A and TFAP2C Expression in Invasive UCC and Invasive SqD** | | | | |
| --- | --- | --- | --- | --- |
| **TFAP2A** | **N** | **Mean** | **STDEV** | **p value** |
| Invasive UCC | 83 | 1.16 | 2.01 | <0.001 (Wilcoxon rank sum) |
| Invasive SqD | 21 | 4.76 | 2.32 |  |
|  | | | | |
| **TFAP2C** | **N** | **Mean** | **STDEV** | **p value** |
| Invasive UCC | 84 | 3.1 | 2.99 | 0.015 (Wilcoxon rank sum) |
| Invasive SqD | 20 | 4.85 | 2.85 |  |
