# Supplementary material for: Repression of transcription factor AP-2 alpha by PPARγ reveals a novel transcriptional circuit in basal-squamous bladder cancer
Source: Oncogenesis. 2019 Nov 26;8(12):69. doi: 10.1038/s41389-019-0178-3 (PMC6879593; doi:10.1038/s41389-019-0178-3)
Supplement: Supplementary file 3 — STableS3 [file 41389_2019_178_MOESM3_ESM.docx]

| **Supplementary Table S3: Association of TFAP2A and TFAP2C Expression with Clinical Variables in Bladder Cancer** | | | | |
| --- | --- | --- | --- | --- |
| **Lymph node metastasis** | | | | |
| **TFAP2A** | **N** | **Positive** | **Negative** | **p value** |
| pN0 | 58 | 8 | 50 | 0.049 (Chi square test) |
| pN1-pN3 | 36 | 11 | 25 |  |
|  | | | | |
| **TFAP2C** | N | Yes | No | p value |
| pN0 | 60 | 27 | 33 | 0.635 (Chi square test) |
| pN1-pN3 | 36 | 18 | 18 |  |
|  | | | | |
| **Distant recurrence** | | | | |
| **TFAP2A** | N | Positive | Negative | p value |
| No recurrence | 76 | 13 | 63 | 0.137 (Chi square test) |
| Recurrence | 21 | 7 | 14 |  |
|  | | | | |
| **TFAP2C** | N | Positive | Negative | p value |
| No recurrence | 78 | 32 | 46 | 0.037 (Chi square test) |
| Recurrence | 21 | 14 | 7 |  |
