# Supplementary material for: Repression of transcription factor AP-2 alpha by PPARγ reveals a novel transcriptional circuit in basal-squamous bladder cancer
Source: Oncogenesis. 2019 Nov 26;8(12):69. doi: 10.1038/s41389-019-0178-3 (PMC6879593; doi:10.1038/s41389-019-0178-3)
Supplement: Supplementary file 4 — STableS4 [file 41389_2019_178_MOESM4_ESM.docx]

**Supplementary Table S4: Antibodies used in this study.**

| **Antibody** | **Catalog Number** | **Company** | **Dilution** | **Application** |
| --- | --- | --- | --- | --- |
| TFAP2A | HPA028850 | Sigma-Aldrich | 1:200  1:1000 | WB  IHC |
| TFAP2C | sc-53162 | Santa Cruz  Biotechnology | 1:200  1:1000 | WB  IHC |
| PPARγ | D69 | Cell Signaling | 1:1000 | WB |
| FABP4 | AF3150 | R&D Systems | 1:1000 | WB |
| GAPDH | 14C10 | Cell Signaling | 1:1000 | WB |
| TP63 | Ab124762 | Abcam | 1:2000 | WB |

WB: Western Blot, IHC: Immunohistochemistry
