# Supplementary figures and images for: Repression of transcription factor AP-2 alpha by PPARγ reveals a novel transcriptional circuit in basal-squamous bladder cancer
Source: Oncogenesis. 2019 Nov 26;8(12):69. doi: 10.1038/s41389-019-0178-3 (PMC6879593; doi:10.1038/s41389-019-0178-3)

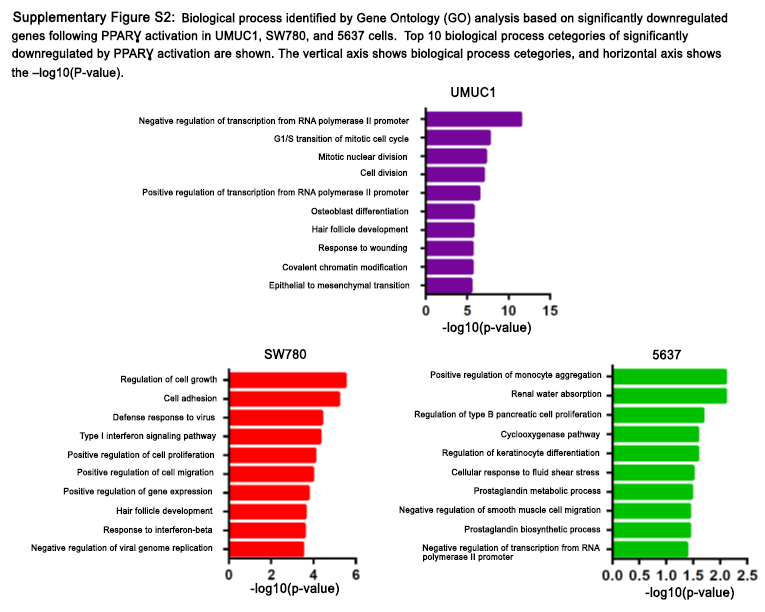

Supplement: Supplementary file 6 — FigS2 [file 41389_2019_178_MOESM6_ESM.tif]
